# “No culture stops me from taking tablets”: Exploring community-level factors influencing pregnant women’s IPTp-SP uptake in Nigeria

**DOI:** 10.1371/journal.pgph.0006745

**Published:** 2026-06-25

**Authors:** Patricia Ogba, Tunde A. Alabi, Andrea Baumann, Norm Archer, Joshua Eniojukan, Bonny Ibhawoh, Deborah DiLiberto

**Affiliations:** 1 Faculty of Health Sciences, Global Health Office, McMaster University, Hamilton, Ontario, Canada; 2 Department of Sociology, University of Lagos, Akoka, Nigeria; 3 Degroote School of Business, McMaster University, Hamilton, Ontario, Canada; 4 Department of Clinical Pharmacy, Niger-Delta University, Wilberforce Island, Bayelsa, Nigeria; 5 Department of History, McMaster University, Hamilton, Ontario, Canada; Jhpiego, UNITED STATES OF AMERICA

## Abstract

Nigeria bears the highest burden of malaria cases in the world, and pregnant women are uniquely vulnerable to the disease. Malaria in pregnancy (MiP) may have negative implications for maternal and neonatal health. The World Health Organization (WHO)-recommended intermittent preventive treatment with sulphadoxine-pyrimethamine (IPTp-SP) helps to prevent MiP. However, due to the influence of significant others, the decision-making power regarding health may be outside the purview of pregnant women. Using the social norms theory, this study investigates the role of community factors affecting the use of IPTp-SP among pregnant women. The study adopted qualitative methods, including focus group discussions with pregnant women and in-depth interviews with significant others, including community leaders, traditional birth attendants, religious leaders, spouses and mothers-in-law. Six focus group discussions and seventeen in-depth interviews were conducted with pregnant women and stakeholders, respectively. Data were managed using NVivo (version 12) and analyzed thematically and deductively in line with the social norms theory. We found that most of the pregnant women demonstrated interest in using IPTp-SP. While they recognized the role of significant others in health decision-making, many expressed agency and resistance against social norms opposing their use of IPTp-SP. They exhibited varying degrees of defiance against domination by spouses and mothers-in-law and gave legitimacy to healthcare professionals. Pregnant women and TBAs adopted mixed approaches (traditional and orthodox medicines) to preventing MiP. Pregnant women deferred to their religious leaders than to their community leaders. For this study’s sample, descriptive norms were more critical. Most women emphasized their will to make health decisions and prioritized their health and their babies, defying the long-standing social norms that militate against women’s health-seeking behaviour.

## Introduction

Malaria remains a significant global health challenge, particularly affecting children and expectant mothers [1–3]. Between 2000 and 2019, annual global malaria cases remained relatively stable, ranging from 227 million to 248 million across 108 endemic countries [4]. However, recent years have seen an upward trend: an estimated 247 million cases were reported in 2021, rising to 252 million in 2022 and 263 million in 2023 [4,5].

Malaria-related deaths declined from 861,000 in 2000–567,000 in 2019, reflecting global progress [4]. However, the COVID-19 pandemic reversed this trend, causing an estimated 55,000 additional deaths in 2020 alone [2,5]. Impressively, mortality has since declined, with 597,000 deaths recorded in 2023 [1,4]. Despite these global gains, the WHO African Region continues to carry the highest burden of malaria-related mortality, accounting for 95% of all global malaria deaths in 2023 and 2024 [1,4]. In 2024, more than half of these deaths occurred in five countries, including Mozambique (3.6%), Ethiopia (4.4%), Uganda (4.7%), the Democratic Republic of Congo (12.5%), and Nigeria, bearing the highest burden of 24.3% (1). About 97% of the total Nigerian population remains at risk of Malaria, leading to an estimated annual economic loss of 132 billion naira – about 86,000 United States Dollars [6]. Pregnant women are particularly vulnerable to malaria due to the reduced immunity associated with pregnancy [2,3,7,8].

MiP poses substantial risks to both maternal and foetal health [2,4,5,9,10]. MiP contributes to maternal anemia, morbidity, and mortality and causes placental sequestration of parasites, which restricts nutrient and oxygen transfer to the fetus. These disruptions can result in stillbirth, preterm birth, intrauterine growth restriction, and low birth weight—adverse outcomes associated with long-term infant morbidity and poor cognitive development [2,4,8–11]. In 2023, approximately 36 million pregnancies occurred across 33 moderate-to-high transmission countries in the WHO African Region; 12.4 million (34%) were infected with malaria [4].

In Nigeria, MiP complicates between 8.4% and 58.1% of pregnancies and contributes to around 11% of maternal deaths [10,11]. Nearly 90% of Nigeria’s 7.5 million annual pregnancies are at risk [10].Malaria accounts for about 50% of reported illnesses and 15% of anemia cases among pregnant women in hospitals [9]. Although reported MiP cases declined from 480,000 in 2017–373,000 in 2019, the burden remains high and demands sustained attention and action [9,12]. To mitigate these risks, the WHO recommends intermittent preventive treatment in pregnancy with sulfadoxine-pyrimethamine (IPTp-SP) as a core component of antenatal malaria prevention [4,5].

IPTp-SP is an entire therapeutic course of antimalarial medicine given to pregnant women monthly from the second trimester until delivery, regardless of a woman’s malaria status [7,13–15]. In Nigeria, IPTp-SP is supposed to be provided free of charge during antenatal care (ANC) as directly observed therapy [7,16,17]. Evidence confirms its efficacy in preventing malaria and its associated complications [14,18,19]. Despite progress in expanding IPTp-SP access globally over the last two decades, uptake remains suboptimal in many African countries, including Nigeria [9,20]. In Nigeria, the proportion of pregnant women receiving at least one dose increased from 27% in 2013 to 64% in 2018. However, uptake of the recommended three doses remains low [21], as only 17% of ANC attendees completed the full regimen in 2018 [22]. Although the Nigerian government has implemented several initiatives to promote IPTp-SP, the country still records one of the highest burdens of MiP. A key barrier is poor adherence to IPTp-SP protocols [7,17,23,24].

Several studies have examined the health system and individual-level factors influencing pregnant women’s IPTp-SP uptake in Nigeria [2,10,14,16,18,24–28]. However, the level of agency to make health decisions across spaces and between rural and urban areas is unclear [29]. Essentially, in rural settings, health decisions may not be wholly dependent on the pregnant woman but also on cultural norms and significant others in the community. However, there is scant evidence on how community-level factors influence IPTp-SP uptake, particularly in socioeconomically disadvantaged settings. Contextual factors can include family relationships, religion, ethnicity, history, traditional beliefs, and cultural values embedded within a community. Contextual dynamics play a critical role in determining the availability, accessibility, and utilization of health interventions. They influence not only supply chains but also individual decision-making, compliance, and trust in health services [30,31].

Guided by Social Norms Theory (SNT), this study investigates (1) community-level factors, including cultural beliefs and family history, that influence pregnant women’s use of IPTp-SP, and (2) the role of significant others (spouses, mothers-in-law, traditional birth attendants, religious and community leaders) in shaping pregnant women’s decisions to use IPTp-SP. To the best of our knowledge, there is no study applying the SNT to IPTp-SP uptake among pregnant women in Nigeria. By exploring the social structures, norms, and beliefs embedded within communities, the study seeks to identify both community barriers and facilitators to IPTp-SP uptake. These insights are essential for informing culturally responsive, community-based strategies to improve malaria prevention in pregnancy.

### Theoretical framework

SNT was first introduced by Perkins and Berkowitz (1986) in the context of addressing excessive alcohol consumption among U.S. college students [32,33]. The theory posits that perceived social norms, that is, what people believe others think and do, shape individual behaviour rather than actual norms. Often, these perceptions are inaccurate [33–35]. A fundamental aspect of SNT is that individuals tend to modify their behaviour to align with what they believe is socially acceptable or common among their peer group. When these perceptions are distorted, they can reinforce harmful behaviours or suppress healthy ones [34,35]. SNT distinguishes between two key types of social norms: descriptive and injunctive norms. Descriptive norms relate to the prevalent behaviour in a typical situation, while injunctive norms refer to the perceptions of what others approve or disapprove [32]. Injunctive norms reflect “what kind of behaviour is approved or disapproved by the community and thereby motivate actions through the anticipation of social rewards or punishment” [36]. Interventions grounded in SNT aim to shift behaviour by revealing the true norms [35] and the extent to which people are able to withstand social pressure when the true norms pose health risks.

This theoretical lens is especially relevant for understanding pregnant women’s uptake of IPTp-SP in Nigeria. While knowledge and access are essential, they are insufficient to ensure pregnant women’s utilization of IPTp-SP. Pregnant women are often influenced by perceived expectations of significant others, including their partners, religious and community leaders. Misperceptions, such as assuming few pregnant women use IPTp-SP or that relevant stakeholders disapprove it, can deter uptake, even when services are available. Applying SNT will allow an exploration of (1) cultural factors that shape women’s uptake of IPTp-SP, and (2) the perception of the influence of significant others and their decision to use IPTp-SP in Nigeria.

## Methods

### Ethics statement

The study was approved by the Hamilton Integrated Research Ethics Board, Canada, with approval number HiREB 14382. An approval was also received from the Bayelsa State Ministry of Health with approval number BSHREC/Vol. 1/21/12/001. Furthermore, community entry was done in each community, and the community leaders gave verbal approval to conduct the study. In addition, informed consent was obtained from each participant before they participated in the study. For the participants who lacked formal education, the research assistants explained the consent form, originally written in English, to them in their local dialects. Upon agreeing to participate, they endorsed the consent forms with their thumbprints. However, the formally educated participants independently read the consent forms, sought clarifications by posing questions, and subsequently affixed their signatures. All the principles of human research ethics, including anonymity, confidentiality and voluntary participation, were upheld.

### Study design

The current study is part of a larger qualitative study investigating multi-level factors influencing the acceptance and utilization of preventive treatments during pregnancy in Nigeria. The current study reports the community-level factors influencing the use of IPTp-SP. The study adopted the in-depth interview (IDI) and focus group discussion (FGD) approaches of data collection. At the start of each focus group, participants were shown a sample of Fansidar (SP). Across all the FGD participants, only three pregnant women, who reported occasional visits to urban health facilities, recognized the medication. Following a brief introduction by the first author (PO), several women recalled purchasing the drug from local chemists after it had been prescribed at community clinics, while others reported that their traditional birth attendants (TBAs) had advised them to buy it directly.

### Study location

The study was conducted in two rural communities (Yenaka and Sabagreia) in Bayelsa State, the south-south region of Nigeria. Bayelsa’s environment is swampy and waterlogged [37], which makes it a suitable habitat for female Anopheles mosquitoes that transfer the malaria-causing parasites into the human bloodstream through bites. In addition, Bayelsa ranked seventh among Nigerian states with the least utilization of the insecticide-treated nets, with a 20.1% utilization rate [38]. This makes the residents more vulnerable to malaria. In addition, Bayelsa is among the ten states with the lowest rate of facility delivery, with 80% of pregnant women delivering outside the hospital [29]. This suggests their low level of attendance at antenatal clinics [39], where they would have been exposed to the WHO-approved IPTp-SP.

Yenaka and Sabagreia are rural communities in Bayelsa State characterized by high poverty levels and limited access to formal health care. In Sabagreia, two cottage hospitals staffed mainly by Community Health Extension Workers (CHEWs) operate only for restricted hours, limiting their availability for routine and emergency care. Yenaka is served by a single primary health centre that faces persistent shortages of trained personnel, essential medicines, and functional medical equipment.

These systemic resource constraints substantially undermine the delivery of maternal and primary health services in both communities. Consequently, many residents rely on traditional and non-conventional forms of care, including herbal remedies and TBAs, rather than public health facilities. This health-seeking pattern was reflected in the present study, as all pregnant participants were recruited through TBA-run antenatal clinics rather than through government health facilities. In addition to TBA services, most pregnant women in this study visited chemists or pharmacies; only three mentioned visiting a hospital occasionally outside the community during their current pregnancy. They visited the community clinics only a few times and were disappointed by the lack of personnel and resources. Further information about the study location and sociocultural context has been published elsewhere [40].

### Study population

Recall that the SNT focuses not only on the actors but also on significant others whose behaviours can shape the actors’ decisions. Hence, the study population comprises (1) pregnant women (primary population), and (2) spouses of the pregnant women, (3) mothers-in-law, (4) religious leaders, (5) community leaders and (6) traditional birth attendants (TBAs) (secondary population). The major inclusion criteria for the entire population were being an adult (aged 18 or above) and being a member of the selected community. Being pregnant was a requirement for the first population. Table 1 summarizes the demographic characteristics of pregnant women. We did not collect detailed socio-demographic information from community stakeholders because they are a secondary population. Additionally, asking respected community stakeholders, such as community leaders, for certain socio-demographics, such as age, may be considered disrespectful by some participants. The mean age of pregnant women was approximately 27. Half of them had no formal education; 36.1% had primary education, while 13.9% had secondary education. An overwhelming majority (97.2%) of them were fish farmers, while one person described herself as a “full housewife.” All the participants, including the stakeholders, identified as Christians.

**Table 1:** Demographic characteristics of pregnant women.

ID	Community	Age	Education	Marital Status	Occupation
PW1	Yenaka	19	None	Unmarried	Farmer
PW2	Yenaka	22	Primary	Unmarried	Farmer
PW3	Yenaka	24	None	Married	Farmer
PW4	Yenaka	27	Secondary	Married	Farmer
PW5	Yenaka	30	Primary	Married	Farmer
PW6	Yenaka	18	None	Unmarried	Farmer
PW7	Yenaka	21	None	Unmarried	Farmer
PW8	Yenaka	25	Primary	Married	Farmer
PW9	Yenaka	28	None	Married	Farmer
PW10	Yenaka	31	Secondary	Married	Farmer
PW11	Yenaka	23	None	Unmarried	Farmer
PW12	Yenaka	26	Primary	Unmarried	Farmer
PW13	Yenaka	29	None	Married	Farmer
PW14	Yenaka	34	Primary	Married	Farmer
PW15	Yenaka	38	Secondary	Married	Farmer
PW16	Yenaka	20	None	Unmarried	Farmer
PW17	Yenaka	22	None	Unmarried	Farmer
PW18	Yenaka	24	Primary	Married	Farmer
PW19	Sabagreia	27	None	Unmarried	Farmer
PW20	Sabagreia	33	Primary	Married	Farmer
PW21	Sabagreia	35	Secondary	Married	Farmer
PW22	Sabagreia	19	None	Unmarried	Farmer
PW23	Sabagreia	23	Primary	Unmarried	Farmer
PW24	Sabagreia	28	None	Married	Farmer
PW25	Sabagreia	32	Primary	Married	Farmer
PW26	Sabagreia	36	Secondary	Married	Farmer
PW27	Sabagreia	21	None	Unmarried	Farmer
PW28	Sabagreia	26	Primary	Married	Farmer
PW29	Sabagreia	29	None	Unmarried	Farmer
PW30	Sabagreia	34	Secondary	Married	Farmer
PW31	Sabagreia	18	None	Unmarried	Farmer
PW32	Sabagreia	24	Primary	Unmarried	Farmer
PW33	Sabagreia	27	None	Married	Farmer
PW34	Sabagreia	31	Primary	Married	Farmer
PW35	Sabagreia	22	None	Unmarried	Farmer
PW36	Sabagreia	25	None	Unmarried	Housewife

### Recruitment and sampling

Four trained research assistants recruited the study participants. Recruitment of participants began on March 23, 2022, and ended on April 19, 2022. Given that non-orthodox treatments were popular in the selected communities, 36 pregnant women (18 from each community) were recruited from TBAs using a convenience sampling technique. The recruitment of pregnant women from TBAs reflects the dominant pregnancy care pathway in these communities and shapes women’s access to IPTp-SP and biomedical malaria prevention services. The pregnant women served as a useful link to their spouses, and four of them (two from each community) participated in the study. In addition, two community leaders, four religious leaders, three TBAs and four mothers-in-law participated in the study, putting the total number of participants at 53 (36 pregnant women and 17 significant others). Six FGDs- comprising six participants each- were conducted with the pregnant women. Seventeen in-depth interviews were conducted with the significant others. The participants represented different tribes, including Eppie, Isoko, Ikorobase, Atia and Ijaw, among others, showing the relative cultural diversity in the communities. Further information about the recruitment and sampling is available [40].

### Research instrument and data collection procedures

Data collection spanned two months from May 9 to June 29, 2022. Data were collected face-to-face. The study utilized structured guides, which were adapted from a similar study [41], for the IDIs and FGDs. The research instruments contained questions regarding attitudes and behaviour of the participants toward the use of IPTp-SP, their normative beliefs, subjective norms related to the utilization and (dis)connections between behaviour intentions and actual use of IPTp-SP. The pilot study was conducted with 10 pregnant women from two contiguous communities to test the appropriateness of the instruments. The participants for the pilot study are excluded from the current analysis. The FGDs were conducted in Ijaw (the common local dialect) or pidgin English, as agreed by the FGD participants. The significant others opted for Ijaw, except for the community leaders, who preferred to be interviewed in English. The interviews with stakeholders lasted 30–46 minutes, while the FGDs with pregnant women lasted 60–94 minutes. Details about the data collection procedures have been published earlier [40].

Data saturation was determined through an iterative process of concurrent data collection and analysis. Saturation was reached when no new codes or themes emerged from subsequent interviews, and the data became repetitive. Specifically, saturation was realized after five FGDs and 16 individual interviews with community stakeholders. The sixth FGD and the seventeenth interview did not yield significantly novel information, indicating that data saturation had been reached.

### Data analysis

All interviews were digitally recorded. The ones conducted in Ijaw and pidgin English were translated into standard English by two native speakers who are also fluent in pidgin and standard English. All interviews and FGDs were transcribed in Microsoft Word and exported to Nvivo (version 12). A community member was hired to cross-check the audio files and transcripts. The initial coding was done by the first author and validated by the second author. To ensure inter-coder reliability, both authors engaged in regular discussions to compare coding outputs, resolve discrepancies, and reach consensus on code definitions and theme categorization. A refined codebook was developed through this process to ensure consistency in applying deductive codes derived from the SNT and inductive codes that emerged within each parent category. This consensus-building process strengthened the trustworthiness of the data analysis. We primarily adopted a deductive coding method in line with the SNT. We organized the codes into descriptive and injunctive norms. We later adopted an inductive coding method within each major parent code.

### Positionality and reflexivity

The first author’s professional background as a registered nurse and midwife with over nine years of experience in Nigerian healthcare settings has provided an in-depth understanding of the systemic challenges surrounding malaria in pregnancy. The fifth author is a male public health scholar in Nigeria and has lived in the state where the study was conducted. While this insider perspective facilitated rapport-building and contextual interpretation, it also had the potential to introduce bias. To mitigate this, we engaged in ongoing reflexive journaling to document assumptions, decisions, and emerging interpretations throughout the research process.

In addition, collaboration with the second author, a sociologist in Nigeria, helped to reduce potential bias and carefully navigate the assumption of the SNT. Data coding and theme validation were conducted collaboratively to ensure analytical rigor and minimize subjective influence.

## Result

The results are divided into three sections: (1) the views of pregnant women regarding descriptive norms and use of IPTp-SP, (2) pregnant women’s views about injunctive norms and use of IPTp-SP from the perspective of pregnant women, and (3) the views of significant others about pregnant women’s use of IPTp-SP. Significant others are people who have a profound influence on pregnant women’s reproductive health-seeking behaviour, including spouses, family members and TBAs.

The first subsection on descriptive norms focused on pregnant women’s use of IPTp-SP and their compliance with or resilience to perennial traditions. Although it discusses the influence of culture, tradition, ethnicity and family history (elements of injunctive norms), the focus and lesson the women’s perception and use of IPTp-SP despite these influences. The second subsection on injunctive norms focuses on the pregnant women’s view on the influence of significant others in their use of IPTp-SP. The third subsection on significant others discusses the views of stakeholders, including spouses, other family members, community leaders and TBAs.

1. **The views of pregnant women- descriptive norms**

This section is divided into two and rests mostly on the idea of descriptive norms. The first section discusses cultural and traditional practices. The second section discusses the influence of ethnicity, and family history.

### Influence of cultural practices – pregnant women’s view

Pregnant women were probed on the influence of traditional and cultural practices on their uptake of IPTp-SP. One of the participants noted that, previously, pregnant women were encouraged to only use herbs and restricted from taking tablets. She admitted that such cultural practices and beliefs no longer carry weight, as it is now well-known that most drugs are produced from herbs. On this premise, she submitted that any culture that currently discourages pregnant women from taking Fansidar should be abolished. Another participant narrated how their cultures encourage women to eat certain foods, like snails and plantain, especially in the 9^th^ month of pregnancy. It is believed that such foods help to ease delivery. She, however, could not identify any cultural beliefs that prohibited Fansidar uptake. Some other pregnant women also stated that cultures banning pregnant women from using Fansidar no longer exist. However, they expressed the willingness to disobey such cultural beliefs and practices if they existed. For example, a participant explained that, “*before this time, there were such laws where herbs were used, that is why tablets were stopped…no culture stops me from taking tablets. Even if there is one, I will not follow it…” (Participant 4,* ***FGD 3****)*.

A few, however, expressed the contrary, stating that they will abstain from Fansidar in favour of traditional herbs if their culture supports only the use of herbs during pregnancy. In this regard, one participant narrated thus: “…*if there is any culture stopping pregnant women from taking Fansidar or any other malaria drugs, I will fully obey and abstain from taking the drugs…(Participant 4,* ***FGD 1****)*.

Regarding tradition, some pregnant women described how some family traditions encouraged using only herbal mixtures during pregnancy. Others explained how their mothers and mothers-in-law often took them, when their delivery dates were close, to traditional houses where they were given things to use, eat and drink to make their delivery easy and fast. The study participants did not mention the specific places and the items offered. However, they opined that the onus lay on them to reject or accept going to such places and using such materials, stating that none of these traditions could stop them from using Fansidar.

### Influence of Ethnicity and Family History – pregnant women’s view

This study’s participants represented various ethnic groups within and outside Bayelsa. None of the participants could identify any current aspect of their ethnicities influencing or discouraging the uptake of Fansidar during pregnancy. Furthermore, most pregnant women could not identify any history in their communities or families that encouraged or discouraged the use of Fansidar. Only one drug was mentioned by one participant: chloroquine injection. Many years ago, a pregnant woman in her family lost her pregnancy after receiving a chloroquine injection. Her family members believed that the drug’s side effects caused the miscarriage. This drug was prohibited in her family due to this historical trend. Aside from that, none of the participants could identify a family or community historical pattern that influences the use of Fansidar. In this regard, a participant reported that “*my ethnic tribe does not stop me from taking tablets during pregnancy…No history in my family/community prohibits pregnant women from taking prescribed tablets*” (*Participant 2, FGD 3*).

2. **The views of pregnant women- injunctive norms**

This section discusses the pregnant women’s views about the role of the significant others (spouses, mothers-in-law, community and religious leaders and TBA) in their uptake of IPTp-SP. The section focuses on injunctive norms and interrogates how the (perceived) beliefs and behaviours of significant others shape IPTp-SP uptake among pregnant women.

### Influence of Spouses – pregnant women’s views

Several women mentioned informing their husbands before using any drug as a form of feedback, but not to seek consent. Almost all the participants said they would use the drug even if their spouses did not consent. In this regard, a participant narrated thus:

*…I will inform my husband about the drug prescribed when I get home but this does not mean that he will decide whether or not I am to use it. I will take it regardless of his opinion because the hospital will only ask me to take what is beneficial for my baby and me (Participant 1, FGD 4)*

As a unique form of defiance, one pregnant woman stated that she would refrain from using Fansidar at her husband’s request so that she could become ill with malaria. This would require her husband to spend more money on treating her rather than spending less on preventing MiP with Fansidar. As she narrated:

*… if he refuses I take Fansidar, then I will not take it. I will not take it so I can fall sick with malaria, so my husband will now pay more to get me treated since he would not allow me to prevent it with Fansidar (Participant 6, FGD 5)*

The quote above shows that obedience to the husband or submission to the traditional gender norms was an indirect form of protest.

Some women were confident that their husbands would agree to their use of Fansidar, citing how their husbands usually motivate them to attend ANC and use all drugs prescribed at the clinic.

*My husband motivates me to go to the hospital for ANC because he does not want any harm to my baby and me. He also encourages me to use whatever drug I m given in the hospital. Additionally, he follows up with me to ensure that I use the medications given to me in the hospital (Participant 3, FGD 6)*

Contrarily, a participant, while resisting traditional gender norms and demonstrating her own will, verbalized that her phobia of all tablets would not allow her to use Fansidar even if her husband supports it. She noted thus:

*My husband’s opinion is essential, but I would still not use Fansidar if he allowed me because of my phobia of tablets… (Participant 2, FGD 5)*

Although the woman experienced fear of possible side effects in the use of Fansidar, she demonstrated a willingness to resist gender norms. She showed that she has the agency to decide what (not) to use.

### Influence of Mothers-in-law – pregnant women’s views

The pregnant women also testified that the opinion of mothers-in-law is usually crucial in marriages in their communities. They narrated how some mothers-in-law discourage their pregnant daughters-in-law from using prescribed drugs. Instead, they take them to spiritual homes for herbs. However, many of the participants in this study stated that they would not accept such herbal blends and would not allow their mothers-in-law to deter them from using medications prescribed by HCPs, particularly Fansidar. For example, a participant stated thus:

*My mother-in-law cannot influence me, especially if she doesn’t want me to use Fansidar. If she tells me not to use it, I will use it without her knowing (Participant 4, FGD 4)*

However, some participants said that they would follow their mother-in-law’s advise to not use Fansidar. A participant narrated thus:

*I won’t take Fansidar if she tells me not to take it(Participant 2, FGD 6)*

### Influence of TBAs – pregnant women’s views

The study recruited pregnant women from traditional homes. This highlights the significance and regard the pregnant women in this community attribute to the care provided by TBAs. Most pregnant women admitted to patronizing TBAs for massage and traditional herbs. They, however, expressed that they would not allow TBAs to deter them from using Fansidar during pregnancy. Despite seeking the services of TBAs for massage and herbs, most study participants acknowledge that TBAs do not possess the same knowledge as doctors and, therefore, would not deter them from using Fansidar. Other participants expressed a willingness to use effective herbs prescribed by a TBA and Fansidar simultaneously.

However, some participants mentioned their lack of belief in the effectiveness of herbal remedies provided by TBAs, indicating that TBAs cannot advise them against the use of Fansidar.

*I can go to a TBA to be massaged but she will not stop me from using Fansidar because I know that she does not know what a doctor knows (Participant 1, FGD 4)*

*Although some herbs are ineffective, quite a few are effective. If my TBA prescribes effective ones, I will use them and Fansidar together (Participant 2, FGD 4)*

*although I visit TBAs for them to massage me, I do not use their herbs because Idon’t believe in them. That is why a TBA cannot advise me against using Fansidar (Participant 6, FGD 4)*

### Influence of Community Leaders and Religious leaders – pregnant women’s views

The study participants recognized and admitted the vital role played by community leaders in guiding them and guarding their cultures and traditions. Most study participants stated that they are not required to follow their community leader’s instructions regarding the use or refusal of Fansidar. In this regard, a participant said, *“…my community leader cannot tell me what to take or not take. They don’t know more than the HCPs who prescribed the drug in the first place. I am not obligated to follow any community leader’s instructions*…” (*Participant 3,*FGD 5). However, a few noted the opposite. They insisted that community leaders, by their position, have access to some information, including details about Fansidar, that could be detrimental to them and their pregnancies. Hence, they said they would obey their community leaders if they asked them to refrain from Fansidar.

Most participants expressed their respect for their religious leaders, who can pray to God on their behalf to avert different illnesses and dangers. All the participants were in sync with their pastors praying for them against malaria. However, they were not on the same page regarding their pastor’s influence on their uptake of Fansidar. About half of the pregnant women said they would disobey their pastor’s instructions not to use Fansidar, because they believe that prayer alone is insufficient to protect them from malaria.

*My pastor cannot discourage me from using Fansidar. I will accept if he volunteers to pray for me so I don’t fall sick with malaria. But after the prayer, I will go ahead and use Fansidar (Participant 5, FGD 3)*

The other half said they would not use Fansidar for malaria prevention if their pastors asked them not to use it after prayers.

*I believe in my pastor’s prayer. If he prays for me and tells me not to use Fansidar, I won’t use it (Participant 1, FGD 1)*

A few participants from the latter said their obedience to their pastor’s instructions would depend on whether they felt sick after their pastor’s prayers. In this regard, a participant explained thus:

*If he tells me not to use Fansidar and prays for me and I don’t feel sick, then I will not use Fansidar (Participant 2, FGD 5)*

The quote above suggests that pregnant women are willing to use Fansidar following their pastor’s blessings. If the pastor discourages them from using it, he must demonstrate that his prayer alone can heal and make them strong. Although all the pregnant women said they respect their pastors, half said they would go ahead and use Fansidar without their pastor’s blessings. An important point here is that pregnant women have deference to religious leaders than to community leaders.

### Influence of Friends, Parents, and Siblings – pregnant women’s views

Many participants admitted that friends are essential and could play crucial roles in influencing one’s health-seeking behavior. The participants stated that they would be careful in following the advice of friends, especially when using malaria preventives during pregnancy. The majority said that no friend of theirs would be able to discourage them from using Fansidar. A participant stated thus:

*I know that people can be influenced by their friends, but none of my friends can stop me from taking a drug prescribed by my doctor…(Participant 5, FGD 3)*

When probed on the influence of parents and siblings, study participants expressed divergent views on the influence of their family members on their uptake of Fansidar. While many mentioned that their parents and siblings could not influence their uptake of Fansidar, a group of women expressed that their family members could influence their decision to take Fansidar. The participants cited various reasons why their family members could influence them. Some said their respect for their parents would make them obey all their instructions, while others expressed that they believe their family members’ advice comes from personal experiences. For example, some pregnant women said that their sisters and mothers advising them not to use Fansidar could be because they (the family members) reacted adversely to it when they used it. A participant narrated thus:

*due to the respect I have for my parents, if they tell me not to take Fansidar, I will not take it (Participant 4, FGD* ***3***)

The quote above shows that friends cannot directly discourage pregnant women from taking Fansidar, but their parents may have some influence over them regarding its use.

3. **The views of significant others- injunctive norms**

This section presents the views of stakeholders (spouses, family members, community leaders and TBAs) about factors shaping pregnant women’s use of IPTp-SP. Understanding these injunctive norms is important for policy intervention because, aside from pregnant women’s agency, the views and perceptions of significant others can help develop culturally specific programs aimed at improving the use of IPTp-SP. The first subsection discusses the perceived influence of financial status, which the second shows the role of culture, tradition, ethnicity and history.

### Influence of financial status

When asked about community factors, significant others highlighted finance as an important factor in IPTp-SP uptake. Considering the country’s economic condition, they acknowledged that financial status could impede pregnant women from accessing IPTp-SP. The participants agreed that finances posed a challenge due to the cost of Fansidar and the need for multiple doses. TBAs shared instances where women who desired to go to the hospital and purchase Fansidar were unable to do so due to financial constraints. Religious leaders narrated families’ struggles in affording basic necessities, making additional expenses for medication a burden. Community leaders explained that in a resource-constrained developing country, men prioritize providing meals for their families over buying medicines with the limited funds available. Understanding the financial difficulties pregnant women face, some mothers-in-law expressed willingness to provide financial aid to their daughters-in-law if necessary. Similarly, TBAs recounted their past support and continuous eagerness to assist their clients financially when needed.

*…with the way the country is, a lack of money can prevent my wife from taking the drug… (****Spouse***)

*…most men, instead of giving their wives one thousand naira to buy Fansidar, would prefer that the one thousand naira be used to feed the whole family… (****Community Leader***)

*… I will try my best to make money available for my daughter-in-law whenever she wants to go to the hospital. (****Mother-in-law***)

### Influence of traditions, ethnicity, cultures, religion, and history

Like the pregnant women, significant others did not think that any aspect of their traditions, ethnicity, cultures, religion, or history would influence pregnant women’s uptake of IPTp-SP. For example, a spouse said that although his tradition does not prohibit pregnant women’s use of prescribed drugs, but even if any aspect of his tradition did, he would ignore it. Another spouse described never letting his pastor advise him on whether his wife should use IPTp-SP. The religious leaders emphasized how the church does not stop anyone from taking prescribed medications because they believe God made all things, including the plants from which drugs are produced.

One religious leader narrated how his church often carried out health outreach programs within the community, during which medical professionals were invited to give health talks to all in attendance. However, one of the religious leaders said that although he would not discourage anyone from taking prescribed tablets, he was happy to pray for and join his faith with any pregnant woman who has faith for healing without using tablets. One community leader thought religious leaders negatively influenced prescribed tablet uptake among pregnant women. He lamented how some religious leaders discourage their congregants from using hospital services, leading to the death of some pregnant women.

*…there is no tradition like that in my place. Even if there are any, I will relocate my wife to another place… (****Spouse***)

*…if a pregnant sister walks up to me and says the doctor gave her some drugs and by faith she does not want to take them, I will follow the sister’s faith and pray for her. (****Religious leader***)

Although the interviewed TBAs do not think traditions could influence pregnant women’s uptake of IPTp-SP, they believe in and prescribe herbs to their clients. One thought pregnant women could use herbs for minor malaria treatment, but a hospital visit and treatment would be needed if the malaria was severe. The other TBA said she usually asked her clients to bathe themselves with bitter leaves, pour oil on their bodies, and spend time under the sun. She never asked them to ingest any of these substances orally. Another TBA said that her approach is to ask pregnant women to drink the water she has blessed. She claimed she does this because she knows everything is in God’s hands. When we asked the TBAs how they usually detected malaria in pregnant women, one said she would ask her client if she had a bitter taste in her mouth. A bitter taste indicates the presence of malaria. Another said she usually checked the pregnant woman’s body temperature and lower eyelids. The colour of the eyelid will indicate whether the woman has malaria.

*They should not drink herbs if malaria is too much, but they should go to the hospital and get drugs to protect themselves (****TBA***)

*…I tell the person with malaria to use bitter leaf to wash herself or pour oil on her body, but not to drink it, and remain in the sun for some time (****TBA***)

*…by checking the person’s eyes…that the corner of the eyes used to be yellow, I will know the person has malaria (****TBA***)

### Discussion

This research adopted a qualitative approach to delve into the community-level contextual factors in Nigeria that influence the use of IPTp-SP for preventing malaria during pregnancy. The study adopted the social norms theory to understand descriptive and injunctive norms shaping the use of Fansidar during pregnancy.

The study highlights the complex interplay between cultural beliefs, traditions, financial constraints, pregnant women’s health-seeking behavior, and the impact of significant others on pregnant women’s IPTp-SP uptake. The study found that although the role of significant others came out strongly, women exhibited resistance against (potential) negative influence and demonstrated agency and a firm decision to use IPTp-SP.

While cultural and traditional practices surrounding herbal remedies and specific foods during pregnancy exist, they do not directly prohibit the use of IPTp-SP among many pregnant women. This finding is consistent with a study conducted in Eastern Nigeria, where pregnant women’s demand for IPTp-SP was not affected by their cultural beliefs [28]. By implication, women’s agency appeared salient for this study’s sample. The finding of this study contradicts the dominant assumptions in Nigeria and Africa that suggest low agency among women and submission to cultural norms [42–44], which makes them do things against their will but to please significant others and tradition.

Although the TBAs did not think traditions influenced IPTp-SP uptake, they acknowledged using herbs for minor malaria treatment. They recommended herbal remedies but advised seeking medical treatment for severe cases. Previous studies have documented this approach, where the TBAs urged their pregnant women clients also to use ANC services at public health facilities [7,45,46]. This finding suggests that TBAs have a dual approach to malaria treatment, incorporating both traditional practices and the recognition of the need for modern medical interventions. However, potential issues can arise from the limitations of TBAs, such as their failure to recognize danger signs, their lack of ability to implement simple, evidence-based interventions for complications, and their tendency to delay referrals when necessary [45]. To tackle the limitations TBAs face, it is crucial to adopt a comprehensive approach that encourages collaboration and integration between TBAs and qualified HCPs [7]. This approach should prioritize establishing transparent and efficient referral systems between TBAs and healthcare facilities.

Further, the study participants do not perceive ethnicity and community history as influential factors affecting the uptake of IPTp-SP among pregnant women. They believe these factors do not directly impact pregnant women’s decision to use IPTp-SP for malaria prevention during pregnancy. This discovery challenges the conclusions drawn in a prior study [47], which indicated an association between pregnant women’s ethnic backgrounds and their compliance with receiving appropriate doses of IPTp-SP. This study suggests that ethnicity and community history may not significantly determine the community’s acceptance and adoption of preventive measures like IPTp-SP.

The influence of significant others, such as husbands and mothers-in-law, varied among the participants. Pregnant women expressed agency and independence in making decisions about IPTp-SP usage, disregarding the opinions of their spouses and resisting interference from their mothers-in-law. This finding aligns with previous studies where pregnant women, though recognizing their husbands’ significant roles in influencing their healthcare access, were ready to defy their husbands’ wishes, if necessary, to prioritize their baby’s health [28,48]. The disregard of husbands’ opinions by many pregnant women, as observed in this study, is unexpected considering the traditional reliance on spousal opinions and financial support for healthcare decisions in Nigeria [13,49–52]. The behavioral change could be linked to the growing economic autonomy of Nigerian women, who now occupy diverse professional roles rather than being solely confined to traditional homemaking duties. For the woman in the study who agreed to her husband’s wish not to use IPTp-SP, she implied that her husband would face the consequences of not supporting her decision to take Fansidar by spending more money to treat her if she fell sick from malaria. Spending more money on treatment may require taking a more powerful anti-malaria and subsequent IPTp-SP use to prevent future MiP. For such women, rather than outrightly disobeying their husbands’ opinions, they resisted implicitly – an indirect form of protest.

In this current study, religious leaders generally supported IPTp-SP usage, emphasizing evidence-based medicine and the compatibility of medicinal plants with their religious beliefs. However, concerns were raised about some religious leaders discouraging their followers from seeking hospital services and relying solely on spiritual treatments. An earlier study reported how pregnant women following the directives of their pastors regarding their health-seeking behaviour have contributed to low IPTp-SP uptake [53]. This finding underscores the need for improved collaboration between HCPs and religious leaders to ensure accurate health information and prevent potential harm [54].

The role of community leaders and TBAs was acknowledged, but their influence on IPTp-SP uptake varied among the participants. Although a portion of the participants showed a willingness to defy the directives of their community leaders regarding the uptake of IPTp-SP, a noteworthy proportion of pregnant women stated their intention to refrain from using IPTp-SP if their community chiefs recommended it. A previous study has documented the influence of community leaders on pregnant women’s uptake of optimal ANC services and IPTp-SP [47].

Further, the majority of the participants acknowledged their reliance on TBAs for services such as massage and the use of traditional herbs. This finding is similar to previous studies, where most pregnant women preferred and utilized TBAs’ services [46,52,55]. However, it is noteworthy that the pregnant women in this current study were firmly determined to use IPTp-SP during pregnancy regardless of any TBA advice. Despite seeking TBA services, the participants recognized the limitations of TBAs’ knowledge compared to medical doctors, emphasizing their autonomy in making decisions regarding using IPTp-SP. This finding highlights the need for collaboration and communication between HCPs and TBAs to ensure a holistic approach to maternal care [7,55,56]. Some participants even indicated a willingness to use both effective herbs prescribed by TBAs and IPTp-SP simultaneously. This finding aligns with a previous study where pregnant women concurrently used TBA services and ANC services in public health facilities [7]. The concurrent use of herbal remedies alongside IPTp-SP is a cause for concern, as it carries the potential for adverse reactions that could jeopardize the health of both the pregnant woman and her unborn child [56–59].

Based on the current study’s findings, pregnant women appear unaffected by their friends’ opinions regarding IPTp-SP uptake. This outcome is consistent with a previous study [60] in which pregnant women stated that their friends did not have the power to influence their medical choices. However, these findings contradict a study conducted in Ghana [46], where participants reported that their friends could sway their decision to seek conventional antenatal care. On the other hand, a significant number of participants in this study acknowledged that they would refuse IPTp-SP if asked to do so by their parents and siblings. This finding aligns with a previous study [46], where their relatives strongly influenced pregnant women’s healthcare-seeking behaviour. The research underscores parents and siblings’ significant influence over pregnant women’s healthcare choices. To address this, HCPs and public health initiatives can prioritize educating both pregnant women and their families about the advantages of consuming IPTp-SP and its role in preventing MiP. By addressing any concerns or misconceptions, it becomes feasible to garner increased support from family members for the adoption of IPTp-SP. This approach aims to create a supportive environment within families, enhancing the likelihood of pregnant women adhering to malaria prevention measures for improved maternal and child health outcomes.

### Limitation

FGDs and IDIs as data collection methods allowed for a comprehensive exploration of participants’ experiences and perceptions, tapping into the richness and depth of their everyday lives. Conducting interviews in Pidgin English, Ijaw, and English based on participants’ linguistic preferences ensured effective communication and understanding. This study, however, has several limitations. First, it was conducted in two rural communities in Bayelsa State, which may limit the transferability of findings to other regions or urban settings with different sociocultural and health system contexts. However, the insights generated provide a valuable, context-specific understanding of factors influencing IPTp-SP uptake in rural Nigerian settings.

Second, some interviews were conducted in Ijaw or Nigerian Pidgin and later translated into English for analysis, introducing the potential for translation bias or loss of nuanced meanings. To minimize this risk, translations were reviewed by bilingual research assistants familiar with the local dialects and context, and the research team engaged in regular discussions to confirm the accuracy and intent of translated expressions.

Third, the cross-sectional design limits the ability to assess attitude–behaviour consistency over time. Longitudinal studies could provide deeper insight into how women’s beliefs and intentions regarding IPTp-SP evolve across pregnancy and influence actual preventive health behaviors.

## Conclusion

This study has shown evidence of women’s behavioural change from being submissive to social norms and the desires of significant others to having agency and being willing to defy any social norms militating against their use of preventive medicines for malaria. This is essential given that the pregnant women were recruited from traditional antenatal settings. While this may suggest that pregnant women in urban centres will defy social norms more than their counterparts in our study location, our findings should be interpreted as reflecting the experiences of pregnant women primarily engaged in informal and traditional antenatal systems in under-resourced rural settings.

Of all the significant others, there is evidence that parents, mothers-in-law and religious leaders have more influence on pregnant women than spouses, friends and community leaders. Interventions grounded in SNT, such as community sensitization on IPTp-SP uptake or promoting endorsements from respected community stakeholders, may help correct misperceptions, shift perceived norms, and ultimately encourage IPTp-SP utilization. This study’s findings highlight the importance of fostering collaboration between HCPs and community stakeholders to enhance the uptake of IPTp-SP among pregnant women. Such collaborative efforts can significantly improve maternal health outcomes within the community.
